# *Helicobacter pylori* and Pro-Inflammatory Protein Biomarkers in Myocardial Infarction with and without Obstructive Coronary Artery Disease

**DOI:** 10.3390/ijms241814143

**Published:** 2023-09-15

**Authors:** Jonatan Wärme, Martin O. Sundqvist, Marcus Hjort, Stefan Agewall, Olov Collste, Christina Ekenbäck, Mats Frick, Loghman Henareh, Claes Hofman-Bang, Jonas Spaak, Peder Sörensson, Shams Y-Hassan, Per Svensson, Bertil Lindahl, Robin Hofmann, Per Tornvall

**Affiliations:** 1 Department of Clinical Science and Education, Södersjukhuset, Karolinska Institutet, SE-118 83 Stockholm, Sweden; 2 Department of Cardiology, Södersjukhuset, SE-118 83 Stockholm, Sweden; 3 Department of Medical Sciences, Uppsala University, SE-751 85 Uppsala, Sweden; 4 Uppsala Clinical Research Center, Uppsala University, SE-751 85 Uppsala, Sweden; 5 Division of Medicine, Institute of Clinical Medicine, University of Oslo, NO-0318 Oslo, Norway; 6 Department of Cardiology, Oslo University Hospital, NO-0450 Oslo, Norway; 7 Division of Cardiovascular Medicine, Department of Clinical Sciences, Danderyd Hospital, Karolinska Institutet, SE-182 88 Stockholm, Sweden; 8 Department of Medicine Huddinge, Karolinska Institute, SE-141 86 Huddinge, Sweden; 9 Coronary Artery Disease Area, Heart and Vascular Theme, Karolinska University Hospital, SE-171 76 Stockholm, Sweden; 10 Department of Medicine Solna, Karolinska Institutet, SE-171 76 Stockholm, Sweden

**Keywords:** myocardial infarction, coronary artery disease, MINOCA, *Helicobacter pylori*, inflammation, biomarkers

## Abstract

Myocardial infarction (MI) with obstructive coronary artery disease (MI-CAD) and MI in the absence of obstructive coronary artery disease (MINOCA) affect different populations and may have separate pathophysiological mechanisms, with greater inflammatory activity in MINOCA compared to MI-CAD. *Helicobacter pylori* (Hp) can cause systemic inflammation and has been associated with cardiovascular disease (CVD). We aimed to investigate whether Hp infection is associated with concentrations of protein biomarkers of inflammation and CVD. In a case-control study, patients with MINOCA (*n* = 99) in Sweden were included, complemented by matched subjects with MI-CAD (*n* = 99) and controls (*n* = 100). Protein biomarkers were measured with a proximity extension assay in plasma samples collected 3 months after MI. The seroprevalence of Hp and cytotoxin-associated gene A (CagA) was determined using ELISA. The associations between protein levels and Hp status were studied with linear regression. The prevalence of Hp was 20.2%, 19.2%, and 16.0% for MINOCA, MI-CAD, and controls, respectively (*p* = 0.73). Seven proteins were associated with Hp in an adjusted model: tissue plasminogen activator (tPA), interleukin-6 (IL-6), myeloperoxidase (MPO), TNF-related activation-induced cytokine (TRANCE), pappalysin-1 (PAPPA), soluble urokinase plasminogen activator receptor (suPAR), and P-selectin glycoprotein ligand 1 (PSGL-1). Hp infection was present in one in five patients with MI, irrespective of the presence of obstructive CAD. Inflammatory proteins were elevated in Hp-positive subjects, thus not ruling out that Hp may promote an inflammatory response and potentially contribute to the development of CVD.

## 1. Introduction

Myocardial infarction (MI) in the absence of obstructive coronary artery disease (MINOCA) is a clinical syndrome with myocardial injury in the absence of significant stenosis in any major epicardial vessel [1]. In patients with acute MI, the prevalence of MINOCA has been estimated to be 1–14% [2]. When compared to patients with MI and obstructive coronary artery disease (MI-CAD), MINOCA patients are more likely to be younger and female [2]. MINOCA, as a working diagnosis, is a heterogenous syndrome with a range of different underlying causes [3,4]. Although the long-term prognosis is favorable compared to MI-CAD, MINOCA is still associated with considerable morbidity and mortality compared to the general population [3,5,6,7,8].

Traditional risk factors for cardiovascular disease (CVD) have been described as less prevalent in MINOCA [8,9]. Inflammation as an important pathophysiological process in MI-CAD is well-established [10], but its role in MINOCA has not been described in similarly extensive detail. Studies investigating biomarkers of inflammation in MINOCA, such as C-reactive protein (CRP), have shown ambiguous results. Higher CRP concentrations have been described in patients with MINOCA compared to healthy controls [11]. The concentration of CRP in MINOCA appears to be higher at the index event but also has a more rapid resolution when compared to MI-CAD [12]. However, in patients with a working diagnosis of MINOCA, higher levels of CRP have been associated with myocarditis rather than myocardial ischemia [13]. In a previous investigation of the study group presently utilized, MINOCA was compared with MI-CAD and healthy controls, showing that several protein biomarkers of CVD representing distinct inflammatory pathways provided discriminatory value between the groups [14]. This supports inflammation as a possible driver behind the development of MINOCA, and those pathways could differ from those of MI-CAD.

Initiating factors for different inflammatory profiles in MINOCA and MI-CAD remain to be elucidated. Chronic infections could be an overlooked risk factor. Although results are inconsistent, *Helicobacter pylori* (Hp) infection has been described as a risk factor for the development of atherosclerotic CVD in meta-analyses [15,16,17]. Putative mechanisms for this association include chronic systemic low-grade inflammation, prothrombotic effects, immunological cross-reactivity, dyslipidemia, and local effects by bacterial virulence factors such as cytotoxin-associated gene A (CagA) [18,19,20,21,22,23,24,25]. Of interest, strains of CagA-positive Hp have been particularly associated with MI-CAD [17,26]. However, there is a paucity of data concerning Hp and the development of MINOCA. The current study aims to investigate the seroprevalence of Hp and CagA in MINOCA compared to matched MI-CAD patients and healthy controls. Potential associations between protein biomarkers and Hp status will also be studied, as will differences in these associations between the groups.

## 2. Results

Clinical characteristics of MINOCA, MI-CAD, and controls are displayed in Table 1. MINOCA patients were less likely to be current smokers and have a history of diabetes mellitus or hyperlipidemia when compared to MI-CAD, whereas they had a higher prevalence of chronic inflammatory disease and past thromboembolic events. Compared to control subjects, MINOCA patients had a higher prevalence of most CVD risk factors (smoking, hypertension, hyperlipidemia, and diabetes mellitus).

The prevalence of seropositivity for Hp IgG and CagA IgG for the three groups is reported in Table 1. Hp antibodies had a prevalence of 20.2% and 19.2% in MINOCA and MI-CAD, respectively, compared to 16.0% in the healthy controls. No statistically significant differences were observed. Of those with a positive Hp result, 45.5% also had antibodies against CagA. The proportion of those with Hp and CagA antibodies was numerically higher in MINOCA compared to MI-CAD and healthy controls, at 60.0%, 31.6%, and 43.8%, respectively, but the difference did not reach statistical significance.

**Figure 1:**
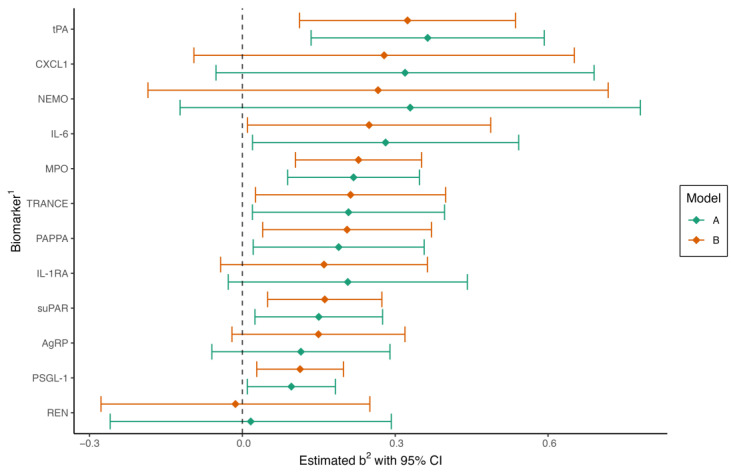
Forest plot of regression coefficients in multiple linear regression for Helicobacter pylori-positive compared to H. pylori-negative patients. Model A is adjusted for age (continuous) and sex. Model B is adjusted for age, sex, group (MINOCA, MI-CAD, or control), body mass index, current smoking, diabetes, hypertension, hyperlipidemia, and eGFR. CI, confidence interval. ^1^ Full names of biomarkers are available in the methods. ^2^ Regression coefficient.

Hp-positive subjects were older than Hp-negative subjects but otherwise non-significantly different in clinical characteristics (Table 2). We found that a total of seven protein biomarkers were associated with Hp serology in the crude regression model A. Regression coefficients for the biomarkers are displayed in Figure 1. All protein biomarkers that were significantly different in the crude model remained so after further adjustment for additional risk factors and group affiliation in model B: tPA (*p* < 0.01), IL-6 (*p* = 0.04), MPO (*p* < 0.001), TRANCE (*p* = 0.03), PAPPA (*p* = 0.02), suPAR (*p* < 0.01), and PSGL-1 (*p* < 0.01). IL-6 and TRANCE were no longer significant after adjusting for multiple tests: tPA (*q* = 0.02), IL-6 (*q* = 0.07), MPO (*q* < 0.01), TRANCE (*q* = 0.05), PAPPA (*q* = 0.04), suPAR (*q* = 0.02), and PSGL-1 (*q* = 0.03).

Hp status and group affiliation as an interaction term were statistically significant in the PSGL-1 model (*p* = 0.04), with a difference in the estimated marginal mean between Hp^+^ and Hp^−^ of 0.08 (95% CI, −0.06–0.22), −0.01 (95% CI, −0.14–0.16), and 0.22 (95% CI, 0.07–0.38) for MINOCA, MI-CAD, and controls, respectively. Hp status was not identified as dependent on group affiliation for the other protein biomarkers. Differences between Hp positive and negative subjects in the crude regression model were most prominent in the control group (tPA, PSGL-1, CXCL1, MPO, IL-1RA, PAPPA, and sUPAR were significantly different). In contrast, no significant differences in biomarkers could be observed between Hp-positive and negative patients in the MINOCA or MI-CAD groups. Associations remained significant in the control group with the adjusted model, and levels of MPO became significantly associated with Hp status in MINOCA patients (Table S1).

## 3. Discussion

In this observational study, we have reported on the serological prevalence of Hp and CagA in patients with MINOCA, MI-CAD, and healthy controls. We have further investigated the relationship between Hp status and protein biomarkers of CVD previously described as differing between the three groups. The main findings were as follows: Approximately one in five patients with MINOCA and MI-CAD were Hp positive, and almost half of those also had evidence of the more virulent CagA strain, but these proportions did not differ significantly when compared with controls. Serological Hp status was significantly associated with seven protein biomarkers previously shown to discriminate MINOCA from MI-CAD or healthy controls. The associations between these seven protein biomarkers and Hp status were not found to depend on the study group, with the exception of PSGL-1.

The difference in seroprevalences of Hp in MINOCA, MI-CAD, and controls was small and not statistically significant. The prevalence of Hp in our study was lower than in a previous smaller study (*n* = 21) on MINOCA patients [27]. This was expected given the period of time elapsed between the studies and a reported decrease in Hp prevalence over time [28]. In a contemporary study of MI-CAD in a Swedish setting, the prevalence of Hp was similar to our results [29]. CagA prevalence in Hp strains varies globally, with a higher prevalence in Asia and Africa. In Europe, slightly above 50% of strains have been reported to carry CagA, which is close to the results in the current study [30]. There was a non-significant trend toward a higher prevalence of CagA in MINOCA compared to both MI-CAD and controls.

Hp-positive subjects were significantly older in comparison to Hp-negative subjects, which is in agreement with previous findings of an increasing prevalence with age [31]. Serological Hp status was significantly associated with seven protein biomarkers of CVD. The largest effects in the adjusted multiple linear regression model were observed for tPA, IL-6, and MPO.

The association between Hp status and plasma concentrations of tPA is a novel finding. Several studies have shown associations between high plasma levels of tPA and incident cardiovascular events, with some suggesting an independent prognostic value [32,33]. tPA is released by endothelial cells in response to thrombus formation, and its best-described function is its role in fibrinolysis by converting plasminogen to plasmin. High levels of tPA are thus not intuitively unfavorable when considering traditional atherosclerotic events caused by plaque rupture. However, basal plasma tPA does not reflect the endothelial ability to release tPA in response to an acute injury but instead indicates endothelial damage with depletion of stored tPA [34]. An alternative explanation could be that Hp can bind plasminogen on its cell surface, leading to a compensatory increase in the production of pro-fibrinolytic substances [35]. However, plasminogen is abundant and not a limiting factor in the process of fibrinolysis, which makes bacterial sequestration unlikely to influence circulating levels of tPA [34]. Hp has previously been associated with other constituents of the fibrinolytic pathway. It has been described to induce local gastric epithelial expression of plasminogen activator inhibitor 1 (PAI-1), urokinase plasminogen activator (uPA), and suPAR, which play important roles in tissue remodeling and are thus important in the neoplastic potential of Hp [36]. The uPA/uPAR system has also been implicated in the atherosclerotic process, with evidence for the adverse effects of suPAR presented both epidemiologically and experimentally [37]. tPA is, in contrast to uPA, specific to the vascular endothelium [34], making local gastric effects unlikely to be the direct cause of the elevated levels shown here.

MPO is a biomarker associated with inflammation and oxidative stress that has been proposed as both a predictor for MI in patients presenting with chest pain as well as an independent risk factor for the development of atherosclerotic CVD [38,39]. Of note, levels of MPO have been found to be higher in MI caused by plaque erosion compared to plaque rupture [40]. Erosions and ruptures of coronary plaques differ in their local immunological environments [41], supporting a theory of distinct underlying pathobiological pathways. The frequency of plaque erosion in MINOCA varies widely in published studies [42,43]. It is nonetheless interesting in this context, given that plaque erosion, like MINOCA, is more common in younger women [44]. Hp proteins stimulate MPO release from neutrophils in the gastric mucosa, and eradication may affect MPO activity [45,46]. These findings not only provide a theoretical link between Hp and CVD but also indicate that treatment of Hp could be beneficial for CVD prevention.

The association between the potent pro-inflammatory cytokine IL-6 and atherosclerotic CVD is well established, and evidence supports its direct causal effect on the inflammation that drives the atherosclerotic process [47]. Traditional clinical risk factors may contribute to the development of CVD, in part through IL-6 [48]. A previous cross-sectional study in healthy subjects investigated correlations between pro-inflammatory biomarkers and serological titers of antibodies against several infectious agents. They found that only Hp was positively associated with IL-6 [49]. The results of the present study support this finding, but caution should be applied given that the association between IL-6 and Hp status only met nominal statistical significance.

The other protein biomarkers that were associated with Hp status are either pro-inflammatory (TRANCE and PSGL-1) or markers of atherosclerosis (PAPPA). Their role in cardiovascular disease is not as well established, but some studies have suggested associations with atherosclerosis [50,51,52]. The association between TRANCE and Hp status should be considered suggestive as it did not meet significance after adjustment for multiple tests.

In a previous analysis of the currently utilized material, tPA, MPO, TRANCE, and PSGL-1 were identified as discriminating between MI-CAD and MINOCA, whereas IL-6, suPAR, and PAPPA differentiated between MINOCA and controls [14]. Here, a positive Hp status was thus associated with protein biomarkers favoring both MI-CAD and MINOCA. The differences in concentrations of protein biomarkers between Hp-positive and negative subjects were most prominent in the control group. It is possible that this finding is due to manifest inflammatory diseases, i.e., MINOCA and MI-CAD, diminishing the difference contributed by Hp. Data on the studied protein biomarkers in MINOCA outside of the current cohort are scarce. The association between Hp and protein biomarkers that were previously shown to have discriminating value for MINOCA against MI-CAD and controls could thus reflect a pathway of inflammation yet to be described in detail.

The study used a well-defined group of MINOCA patients in which care has been taken to exclude differential diagnoses such as myocarditis, cardiomyopathy, and pulmonary embolism. Biomarker analyses in MI present some practical difficulties, as optimal measurement would be performed prior to or after the stabilization of an acute clinical event. In the present study, laboratory samples were collected after the acute phase of the disease, limiting exposure to inflammation induced by the myocardial injury. To our knowledge, this is the first study linking the described biomarkers to Hp in groups with and without CAD.

The study is limited by the size of the study groups. This limits external validity by increasing the risk of both type II errors and chance findings. In order to limit the impact of the latter, we only chose to study biomarkers that had previously been attributed discriminatory value between the study groups. The different etiologies behind the working diagnosis of MINOCA are not fully reflected in the current study; hence, we cannot differ between them. Additionally, the diagnosis has evolved over time, and some of the patients included in the current study, such as those with Takotsubo cardiomyopathy, may not be “true” MINOCA patients by contemporary definitions [4]. MINOCA patients were only included in the current cohort if they had stenosis <30% at coronary angiography, meaning they had less stenosis than MINOCA patients in general. Evidence has also emerged that a thromboembolic ischemic event may be the causative pathophysiology in the majority of MINOCA patients, with a mechanism similar to that of MI-CAD [42]. Thus, transient and partial thrombosis at sites of non-obstructive plaques or erosions could be the cause in a substantial subset of MINOCA patients, with risk factors that overlap those for atherosclerotic CAD. These factors may dilute the findings and limit their generalizability. Serological testing also carries limitations, mainly that it does not allow differentiation between active and prior infections and has a lower sensitivity compared to other available diagnostic tools [53]. This may be due in part to an underestimation in older patients, as IgG titers are lower as gastric lesions caused by the Hp infection progress [28].

## 4. Materials and Methods

### 4.1. Study Cohort

Study subjects were gathered from the case-control study Stockholm Myocardial Infarction with Normal Coronaries, which has been described previously [11]. In summary, the study screened for MINOCA at five coronary care units in Stockholm, Sweden, between June 2007 and May 2011. Inclusion criteria were the age of 35–70 years, fulfillment of the criteria for acute MI, and normal or near normal (<30% stenosis) coronary arteries at angiography. A total of 176 patients were screened. A total of 76 patients were excluded due to a history of structural heart disease or CAD, a pacemaker, severe chronic obstructive pulmonary disease with hypoxemia due to acute exacerbation, severe renal failure, non-sinus rhythm at admission, myocarditis, or pulmonary embolism. A total of 100 patients with MINOCA were ultimately included in the study. For comparison, patients with MI-CAD (*n* = 100) and individuals without symptoms or signs of CAD (*n* = 100), i.e., healthy controls, were also included. These participants were matched to the MINOCA group by age and sex. The control group was randomly selected from the population register in Stockholm by date of birth and sex. They were invited by letter and included in the study after achieving a normal exercise stress test.

All study subjects gave written informed consent at inclusion. This study was conducted in accordance with the Declaration of Helsinki after approval by the Stockholm Ethical Review Board (DNR 2004/4:5).

### 4.2. Laboratory Analyses

Plasma EDTA and citrate plasma were collected at a study visit 3 months after the index event for subjects in the MINOCA and MI-CAD groups and at the study visit for controls. Samples were stored at −80 °C until analysis. Plasma EDTA samples were used to measure protein biomarkers of CVD and inflammation [14]. In brief, 92 biomarkers were measured simultaneously by proximity extension assay technology using the Olink Proseek^®^ Multiplex CVD I^96 × 96^ assay (Olink Proteomics AB, Uppsala, Sweden) and subsequently by real-time polymerase chain reaction with Fluidigm Biomark HD (Standard BioTools Inc, San Fransisco, CA, USA). Results were obtained in log-transformed relative units. A total of 8 patients were lacking plasma samples; thus, the biomarker results of 292 subjects (97 MINOCA, 97 MI-CAD, and 98 controls) were available for analysis. Fourteen of the biomarkers had discriminatory values between MINOCA and MI-CAD or MINOCA and controls [14]. The proposed biological functions of the protein biomarkers were assessed using the Universal Protein Resource [54]. We excluded two proteins associated with myocardial injury as our aim was to evaluate Hp as a risk factor for inflammation and vascular pathology. Accordingly, a total of 12 protein biomarkers were studied: P-selectin glycoprotein ligand 1 (PSGL-1), C-X-C motif chemokine 1 (CXCL1), TNF-related activation-induced cytokine (TRANCE), pappalysin-1 (PAPPA), tissue-type plasminogen activator (tPA), myeloperoxidase (MPO), interleukin-1 receptor antagonist protein (IL-1RA), renin (REN), NF-k-B essential modulator (NEMO), interleukin-6 (IL-6), soluble urokinase plasminogen activator surface receptor (suPAR), and agouti-related protein (AgRP).

Citrate plasma samples were used for serological analysis of Hp IgG using an enzyme-linked immunosorbent assay (ELISA) (Abcam plc, Cambridge, UK) according to the manufacturer’s instructions. A total of 2 patients were lacking plasma samples, leaving 298 subjects (99 MINOCA, 99 MI-CAD, and 100 controls) for analysis. Inconclusive titer results, as specified by the manufacturer, occurred in five subjects. These were regarded as negative after repeat measurements also rendered inconclusive results. In subjects with a positive Hp serology, a subsequent measurement of anti-CagA IgG was performed using an ELISA (Euroimmun Medizinische Labordiagnostika AG, Lübeck, Germany) according to the manufacturer’s instructions. Inconclusive titer results occurred in three subjects and were regarded as negative after repeat measurements.

### 4.3. Statistical Analysis

Continuous variables are reported as medians with interquartile ranges. Categorical variables are described as counts and percentages. For formal testing of continuous variables, the Wilcoxon–Mann–Whitney test was used for comparisons between two groups, and the Kruskal–Wallis test was used for more than two groups. χ^2^ tests were used for comparisons of categorical variables. Baseline characteristics in the study groups were not subjected to formal testing except for Hp and CagA status. Multiple linear regression models were used to assess how Hp status affected the levels of pre-selected biomarkers. Model A (the crude model) utilized Hp status, age, and sex as independent variables, and the biomarkers were used as the dependent variables in separate models for each biomarker. Additional covariates were justified in a directed acyclic graph (Figure S1). In model B, further adjustments were made for group affiliation, BMI, current smoking, diabetes, hypertension, hyperlipidemia, and the estimated glomerular filtration rate (eGFR). The eGFR was calculated using the cystatin C equation from CKD-EPI 2012 [55]. The models were then used on a group basis to determine any differences in regression coefficients. For any protein biomarkers significantly associated with Hp status, Hp status and study group affiliation were used as interaction terms in the linear model to investigate if the effect was dependent on the study group.

R 4.2.1 (The R Foundation for Statistical Computing, Vienna, Austria) and RStudio 2022.07.1 (Posit Software PBC, Boston, MA, USA) were used for the statistical analyses. A *p*-value < 0.05 was considered statistically significant. To adjust for multiple testing, the false discovery rate was controlled at 0.05 using the Benjamini–Hochberg method.

## 5. Conclusions

In this retrospective cohort with samples collected 3 months after an MI event, Hp status had a positive association with seven protein biomarkers of CVD and inflammation. There were no statistically significant differences in seroprevalence of Hp between MINOCA, MI-CAD, and controls. However, our study cannot rule out that Hp may still play a role in the development of MI with and without obstructive CAD, given the association between Hp and inflammatory proteins. Larger epidemiological studies and mechanistic investigations are needed to confirm these results and elucidate potential pathobiological pathways.

## Figures and Tables

**Table 1:** Clinical characteristics by group.

	MINOCA (*n* = 99)	MI-CAD (*n* = 99)	Control (*n* = 100)	Total (*N* = 298)	*p*-Value
*H. pylori* seropositivity	20 (20.2%)	19 (19.2%)	16 (16.0%)	55 (18.5%)	0.727
CagA seropositivity					0.399
CagA^−^	8 (8.1%)	13 (13.1%)	9 (9.0%)	30 (10.1%)	
CagA^+^	12 (12.1%)	6 (6.1%)	7 (7.0%)	25 (8.4%)	
Hp^−^	79 (79.8%)	80 (80.8%)	84 (84.0%)	243 (81.5%)	
Female sex	71 (71.7%)	71 (71.7%)	72 (72.0%)	214 (71.8%)	0.999
Age, years	60.0 (52.0, 65.0)	60.0 (53.0, 65.0)	60.5 (52.8, 65.0)	60.0 (52.0, 65.0)	0.810
Current smoking	21 (21.2%)	33 (33.3%)	7 (7.0%)	61 (20.5%)	<0.001
Hypertension	37 (37.4%)	46 (46.5%)	17 (17.0%)	100 (33.6%)	<0.001
Hyperlipidemia	9 (9.1%)	20 (20.2%)	4 (4.0%)	33 (11.1%)	<0.001
Diabetes	4 (4.0%)	10 (10.1%)	0 (0.0%)	14 (4.7%)	<0.01
Past thromboembolic event or coagulopathy	6 (6.1%)	1 (1.0%)	2 (2.0%)	9 (3.0%)	0.089
Chronic inflammatory disorder ^1^	30 (30.3%)	20 (20.2%)	10 (10.0%)	60 (20.1%)	0.002
BMI	25.8 (22.6, 28.2)	26.3 (23.6, 29.7)	24.6 (23.1, 27.2)	25.4 (23.1, 28.3)	0.017
Hemoglobin, g/L	139 (129, 147)	141 (131, 150)	139 (129, 145)	139 (131, 147)	0.162
eGFR, mL/min/1.73 m^2^	96 (82, 104)	85 (76, 102)	94 (82, 106)	93 (80, 104)	0.048
Cholesterol, mmol/L	5.00 (4.50, 5.70)	5.30 (4.43, 6.00)	5.60 (4.90, 6.40)	5.30 (4.60, 6.10)	0.002
LDL, mmol/L	2.90 (2.50, 3.55)	3.45 (2.70, 4.03)	3.50 (2.90, 4.20)	3.30 (2.70, 3.90)	<0.001
HDL, mmol/L	1.40 (1.20, 1.90)	1.20 (1.00, 1.50)	1.60 (1.10, 1.90)	1.30 (1.10, 1.80)	<0.001
Triglycerides, mmol/L	0.94 (0.78, 1.20)	1.20 (0.86, 1.56)	1.00 (0.76, 1.30)	1.00 (0.78, 1.30)	0.013

Values are expressed as n (%) or median (Q1, Q3). Abbreviations: BMI, body mass index; CagA, cytotoxin-associated gene A; eGFR, estimated glomerular filtration rate; Hp, Helicobacter pylori; HDL, high-density lipoprotein; LDL, low-density lipoprotein. ^1^ Conditions: chronic obstructive pulmonary disorder, asthma, rheumatologic diseases, hepatitis, primary biliary cirrhosis, chronic pancreatitis, diverticulitis, collagenous colitis, and chronic tooth infections.

**Table 2:** Clinical characteristics by *Helicobacter pylori* status.

	Hp^+^ (*n* = 55)	Hp^−^ (*n* = 243)	Total (*N* = 298)	*p*-Value
Female sex	43 (78.2%)	171 (70.4%)	214 (71.8%)	0.245
Age, years	63.0 (56.0, 67.0)	59.00 (52.0, 65.0)	60.00 (52.0, 65.0)	0.040
Current smoking	14 (25.5%)	47 (19.3%)	61 (20.5%)	0.310
Hypertension	22 (40.0%)	78 (32.1%)	100 (33.6%)	0.262
Hyperlipidemia	6 (10.9%)	27 (11.1%)	33 (11.1%)	0.966
Diabetes	2 (3.6%)	12 (4.9%)	14 (4.7%)	0.680
Past thromboembolic event or coagulopathy	1 (1.8%)	8 (3.3%)	9 (3.0%)	0.564
Chronic inflammatory disorder ^1^	15 (27.3%)	45 (18.5%)	60 (20.1%)	0.144
BMI	26.7 (23.7, 29.0)	25.2 (22.9, 28.3)	25.4 (23.1, 28.3)	0.114
Hemoglobin, g/L	136 (130, 145)	139 (131, 147)	139 (131, 147)	0.375
eGFR, mL/min/1.73 m^2^	93 (79, 100)	93 (80, 105)	93 (80, 104)	0.649
Cholesterol, mmol/L	5.30 (4.70, 6.05)	5.30 (4.60, 6.10)	5.30 (4.60, 6.10)	0.574
LDL, mmol/L	3.20 (2.80, 4.00)	3.30 (2.70, 3.90)	3.30 (2.70, 3.90)	0.668
HDL, mmol/L	1.40 (1.20, 1.70)	1.30 (1.10, 1.80)	1.30 (1.10, 1.80)	0.459
Triglycerides, mmol/L	1.10 (0.83, 1.45)	1.00 (0.77, 1.30)	1.00 (0.78, 1.30)	0.535

Values are expressed as n (%) or median (Q1, Q3). Abbreviations: BMI, body mass index; Hp, *Helicobacter pylori*; HDL, high-density lipoprotein; LDL, low-density lipoprotein. ^1^ Conditions: chronic obstructive pulmonary disorder, asthma, rheumatologic diseases, hepatitis, primary biliary cirrhosis, chronic pancreatitis, diverticulitis, collagenous colitis, and chronic tooth infections.

## Data Availability

Metadata is available upon request from the corresponding author. The data are not publicly available due to ethical and privacy concerns.

## Supplementary Materials

The following supporting information can be downloaded at: https://www.mdpi.com/article/10.3390/ijms241814143/s1.
