# Supplementary material for: Helicobacter pylori and Pro-Inflammatory Protein Biomarkers in Myocardial Infarction with and without Obstructive Coronary Artery Disease
Source: Int J Mol Sci. 2023 Sep 15;24(18):14143. doi: 10.3390/ijms241814143 (PMC10531769; doi:10.3390/ijms241814143)
Supplement: Supplementary file 1 [file ijms-24-14143-s001.zip › ijms-2579328-supplementary.pdf]

Table S1. Regression coefficients for Helicobacter pylori^{+} compared to Hp^{-} subjects by group affiliation.

**Biomarker**	**Overall (b[1] [95%CI])**		**MINOCA (b [95%CI])**		**MI-CAD (b [95%CI])**		**Control (b [95%CI])**	
	**Model A**	**Model B**	**Model A**	**Model B**	**Model A**	**Model B**	**Model A**	**Model B**
**tPA**	0.36 (0.13–0.59)	0.32 (0.11–0.54)	0.23 (-0.15–0.60)	0.23 (-0.13–0.59)	0.25 (-0.12–0.62)	0.22 (-0.17–0.61)	0.59 (0.21–0.98)	0.56 (0.17–0.94)
**PSGL-1**	0.10 (0.01–0.18)	0.11 (0.03–0.20)	0.07 (-0.08–0.22)	0.09 (-0.06–0.23)	-0.00 (-0.14–0.14)	0.03 (-0.10–0.17)	0.24 (0.07–0.42)	0.23 (0.06–0.40)
**CXCL1**	0.32 (-0.05–0.69)	0.28 (-0.10–0.65)	0.13 (-0.47–0.72)	0.04 (-0.58–0.66)	0.00 (-0.76–0.76)	-0.09 (-0.87–0.69)	0.89 (0.31–1.47)	0.89 (0.29–1.50)
**TRANCE**	0.21 (0.02–0.40)	0.21 (0.03–0.40)	0.26 (-0.10–0.61)	0.17 (-0.17–0.51)	0.08 (-0.20–0.37)	0.03 (-0.26–0.32)	0.30 (-0.06–0.67)	0.29 (-0.07–0.65)
**MPO**	0.22 (0.09–0.35)	0.23 (0.10–0.35)	0.21 (-0.00–0.42)	0.23 (0.02–0.44)	0.22 (-0.02–0.46)	0.23 (-0.02–0.48)	0.23 (0.02–0.44)	0.24 (0.03–0.45)
**IL-1RA**	0.21 (-0.03–0.44)	0.16 (-0.04–0.36)	0.17 (-0.24–0.57)	0.10 (-0.26–0.47)	-0.01 (-0.38–0.37)	-0.01 (-0.37–0.35)	0.47 (0.05–0.89)	0.39 (0.03–0.75)
**PAPPA**	0.19 (0.02–0.36)	0.21 (0.04–0.37)	0.03 (-0.30–0.36)	0.08 (-0.25–0.41)	0.08 (-0.21–0.36)	0.18 (-0.10–0.45)	0.45 (0.20–0.70)	0.46 (0.21–0.71)
**REN**	0.02 (-0.26–0.29)	-0.01 (-0.28–0.25)	-0.19 (-0.67–0.28)	-0.23 (-0.72–0.24)	0.13 (-0.38–0.64)	0.13 (-0.39–0.65)	0.02 (-0.40–0.44)	0.02 (-0.40–0.45)
**NEMO**	0.33 (-0.12–0.78)	0.27 (-0.19–0.72)	0.42 (-0.35–1.18)	0.40 (-0.38–1.19)	0.19 (-0.70–1.08)	0.25 (-0.68–1.19)	0.37 (-0.36–1.10)	0.32 (-0.41–1.05)
**IL-6**	0.28 (0.02–0.54)	0.25 (0.01–0.49)	0.23 (-0.27–0.74)	0.24 (-0.27–0.75)	0.27 (-0.14–0.67)	0.23 (-0.16–0.62)	0.21 (-0.20–0.63)	0.18 (-0.22–0.59)
**AgRP**	0.11 (-0.06–0.29)	0.15 (-0.02–0.32)	0.04 (-0.27–0.34)	0.08 (-0.22–0.39)	0.09 (-0.24–0.42)	0.10 (-0.23–0.42)	0.25 (-0.04–0.54)	0.26 (-0.02–0.54)
**suPAR**	0.15 (0.02–0.28)	0.16 (0.05–0.27)	0.05 (-0.16–0.26)	0.12 (-0.07–0.31)	0.12 (-0.08–0.33)	0.12 (-0.08–0.33)	0.22 (0.00–0.43)	0.21 (0.01–0.42)

Model A is adjusted for age (continuous) and sex. Model B is additionally adjusted for smoking, BMI, hypertension, diabetes mellitus, hyperlipidemia, group affiliation (MINOCA, MI-CAD, or control), and eGFR. Full names of biomarkers are given in supplementary table A.

BMI, body mass index; eGFR, estimated glomerular filtration rate; Hp, *Helicobacter pylori*; MI-CAD, myocardial infarction with obstructive coronary artery disease; MINOCA, myocardial infarction in the absence of obstructive coronary artery disease.

[1]Regression coefficient

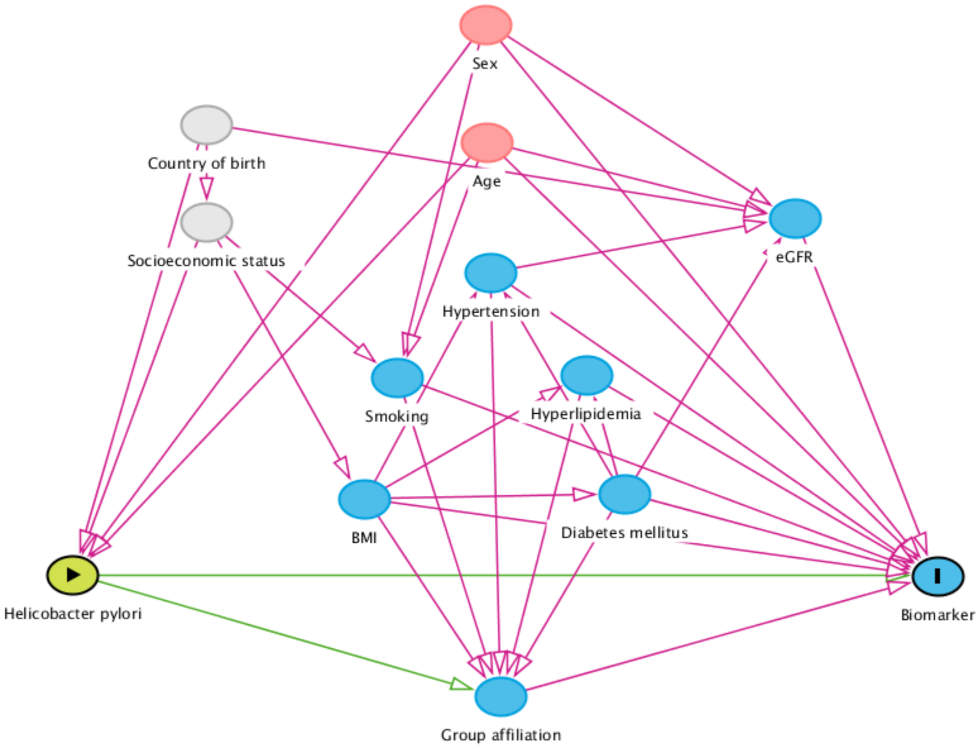

Figure S1. Directed acyclic graph for the association between *Helicobacter pylori* and biomarker concentrations, displaying confounders used in linear regression. Group affiliation concerns MINOCA, MI-CAD, or control. BMI, body mass index; eGFR, estimated glomerular filtration rate; MI-CAD, myocardial infarction with obstructive coronary artery disease; MINOCA, myocardial infarction in the absence of obstructive coronary artery disease.
